# Modeling household dynamics on Respiratory Syncytial Virus (RSV)

**DOI:** 10.1371/journal.pone.0219323

**Published:** 2019-07-09

**Authors:** Wiriya Mahikul, Lisa J. White, Kittiyod Poovorawan, Ngamphol Soonthornworasiri, Pataporn Sukontamarn, Phetsavanh Chanthavilay, Graham F. Medley, Wirichada Pan-ngum

**Affiliations:** 1 Department of Tropical Hygiene, Faculty of Tropical Medicine, Mahidol University, Bangkok, Thailand; 2 Mahidol-Oxford Tropical Medicine Research Unit, Faculty of Tropical Medicine, Mahidol University, Bangkok, Thailand; 3 Centre for Tropical Medicine, Nuffield Department of Medicine, University of Oxford, Oxford, United Kingdom; 4 Department of Clinical Tropical Medicine, Faculty of Tropical Medicine, Mahidol University, Bangkok, Thailand; 5 College of Population Studies, Chulalongkorn University, Bangkok, Thailand; 6 Institute of Research and Education Development, UHS, Vientiane, Lao PDR; 7 Centre for Mathematical Modelling of Infectious Disease & Department of Global Health and Development, London School of Hygiene and Tropical Medicine, London, United Kingdom; Louisiana State University System, UNITED STATES

## Abstract

Respiratory Syncytial Virus (RSV) is the most common cause of respiratory tract infection in infants and children and shows increasing trend among elderly people worldwide. In many developing country settings, population and household structures have gone through some significant changes in the past decades, namely fewer births, more elderly population, and smaller household size but more RSV high-risk individuals. These dynamics have been captured in a mathematical model with RSV transmission dynamics to predict the disease burden on the detailed population for future targeted interventions. The population and disease dynamics model was constructed and tested against the hospitalization data for Acute Lower Respiratory Tract Infection due to RSV in rural Thai settings between 2005 and 2011. The proportion of extended families is predicted to increase by about 10% from 2005 to 2020, especially for those with elderly population, while the classic nuclear family type (with adults and children) will decline by about 10%. For RSV, infections from extended family type (approximately 60% of all household types) have majorly contributed to the force of infection (FOI). While the model predicted the increase of FOI from the extended family by 15% from 2005 to 2020, the FOI contributed by other household types would be either stable or decrease in the same time period. RSV incidence rate is predominantly high among babies (92.2%) and has been predicted to decrease slightly over time (from 940 to 864 cases per 100,000 population by 2020), while the incidence rates among children and elderly people may remain steadily low over the same period. However, the estimated incidence rates among elderly people were twice than those in children. The model predicts that approximately 60% of FOI for RSV will come from members of the extended family type. The incidence rate of RSV among children and elderly in extended families was about 20 times lower than that in infants and the trend is steady. Targeted intervention strategies, such as health education in some specific groups and targeted vaccination, may be considered, with the focus on extended family type. Target interventions on babies can lessen the transmission to children and elderly especially when transmission within households of extended family type is high.

## Introduction

Respiratory Syncytial Virus (RSV) is a communicable disease pathogen, and the most common cause of respiratory tract infection in infants and children [1,2]. It is the most important viral pathogen causing lower respiratory tract infections (LRTIs), leading to pneumonia and bronchiolitis [3]. RSV is transmitted by contact from people who have an active RSV infection. Close contacts within households may present potential opportunities for RSV transmission [4]. The routes of transmission are large-particle aerosols over short distances such as sneezes, hand-to-eye, and by hand contact with infectious secretions, which are passed from the infectious persons to susceptible ones [5–7]. The symptoms include a cough and mild-to-moderate nasal congestion with clear rhinorrhea. At the early stage, a mild fever can occur and symptoms may persist for one to three weeks before complete recovery [8]. The median latency period is 4 days (95% CI 2–8) [5,9], while the infectious period is 4.4 days (95% CI 1–9) [10]. Shedding of the virus and severity of infection may vary with age; for example, adults shed the virus for 3–7 days [11], infants usually shed up to 14 days [12]. They may also vary with immunocompromised status such as for patients with rheumatic diseases, solid tumors [13], and allogeneic transplantation [14]. Moreover, shedding is extended for a significantly longer time in infants with LRTI than in those with clinical manifestations limited to the upper respiratory tract. The immunocompromised people can spread the infection for several months [15]. Duration of waning of short-term immunity of recovered individuals is about 2 years [16–18].

Severe LRTI was estimated at approximately 3 million cases worldwide in 2005 and up to 200,000 deaths among children aged less than 5 years were attributable to RSV [2]. Ministry of Public Health, Thailand, in collaboration with the International Emerging Infections Program of the Global Disease Detection, Thailand Regional Center, launched an active population-based surveillance in two provinces, Sa Kaeo and Nakhon Phanom, in 2003, with the aim to detect RSV-associated acute lower respiratory tract infection (ALRI) through reverse transcriptase polymerase chain reaction (RT- PCR) assays and to describe the burden and characteristics of pathogens that cause pneumonia [19]. The study showed that the incidence of RSV-associated ALRI hospitalization was 46/100,000 persons/year. Overall RSV hospitalization rates were the highest among children aged 1 year (1,067/100,000 persons/year) and 1–4 years (403/100,000 persons/year), but low among enrolled adults aged more than 65 years (42/100,000 persons/year). After 2008, the method of detection has changed to real-time reverse transcription polymerase chain reaction (rRT-PCR) [20]. As the result of higher sensitivity, the overall incidence of RSV-associated ALRI hospitalization was 85/100,000 persons/year. The highest rate occurred among children < 1 year old (1,543/100,000 persons/year). The rates were low among older children and young adults but high among persons aged > 65 years (130/100,000 persons/year). Annual RSV hospitalizations peaked during July-October with almost no documented RSV hospitalizations during January-June.

Thailand is going through a changing phase in the population structure, birth and mortality rates, population growth, household types, household sizes, and migration rates. These changes affect public health decisions and implications for non-communicable [21] and communicable diseases [22]. Many RSV transmission dynamic models have assumed static household distributions. However, household structure has long been known to play an important role in the transmission of infection [22–24]. Disease control measures are often directed at household members, for example, household-based interventions to slow the spread of pandemic influenza [25]. Three household types were targeted in our study, namely:

1. “Nuclear family type 1” (husband and wife or single (aged 15–59 years old) without baby (aged ≤ 1) or children (aged 2–14))
2. “Nuclear family type 2” (husband and wife (aged 15–59) with baby (aged ≤ 1) or children (aged 2–14))
3. “Extended family type” (three-generation family) (husband and wife (aged 15–59) with baby (aged ≤ 1), children (2–14), and grandparents (aged ≥ 60))

In the past 25 years, the extended family type has increased from 25.2% to 33.6% by 2015 and become a dominant family type in Thai society, especially in the rural areas, while the classic nuclear family type 2, a dominant family type in the past, has declined from 52.4% to 26.6% by 2015. Nuclear family type 1 has increased three-folds from 11.7% to 30.1% by 2015 with a slightly greater number and proportion in rural areas compared to the urban [26]. This has reflected in the culture of the country, especially in rural settings, where grandparents often help in looking after their grandchildren while the parents are working away from home.

Many mathematical models have been applied to study RSV transmission dynamics [27–30] but household dynamic was incorporated only recently in the modeling, and only a few of them have looked at disease transmission in general within and between households [22,24]. The studies summarized that household models that include births, deaths, and movement between households could show dramatically different patterns of infection and immunity from static population models. They also suggested that models that did not account for future demographic change and especially its effect on household structure, might potentially overestimate the impact of vaccination. Some previous studies have directly examined the transmission of RSV within households [31,32]. They showed that school-going siblings frequently introduced RSV into households, leading to infection in infants. A recent individual-based model was developed to look at the transmission dynamic of RSV within households in an African setting [33]. The study summarized that it was important to consider the social structuring and size of the household when modeling RSV transmission. The main objective of this study is to study the household dynamics and changing trends of RSV incidence using wild nationwide demographic data of household structuring and size. The model is set up to explore the potential impacts on RSV disease burden in societies that move towards an aging era with a large proportion of the population living in extended families, the common household type for many low/middle income countries. This modeling exercise will be useful for designing target interventions focusing on specific age groups and/or household levels.

## Methods

### Household sub-model

We generated a household deterministic sub-model to calibrate the population in the three household types (see Fig A in S1 File). We classified the households into three major types: nuclear family type 1, nuclear family type 2, and extended family type. We used actual population data of Thailand between 1995 and 2011 from the Population and Housing Census [34,35] and features of Thai families [26] by using the birth and death rates and the population of each household as the initial condition since 1995. All population was transitional with the household transition rate (*hr*) [36], while the death rate was age related [37]. We solved a large set of Ordinary Differential Equations (ODE) of household deterministic sub-model defined in S1 File.

### RSV transmission dynamic model

**Fig 1:**
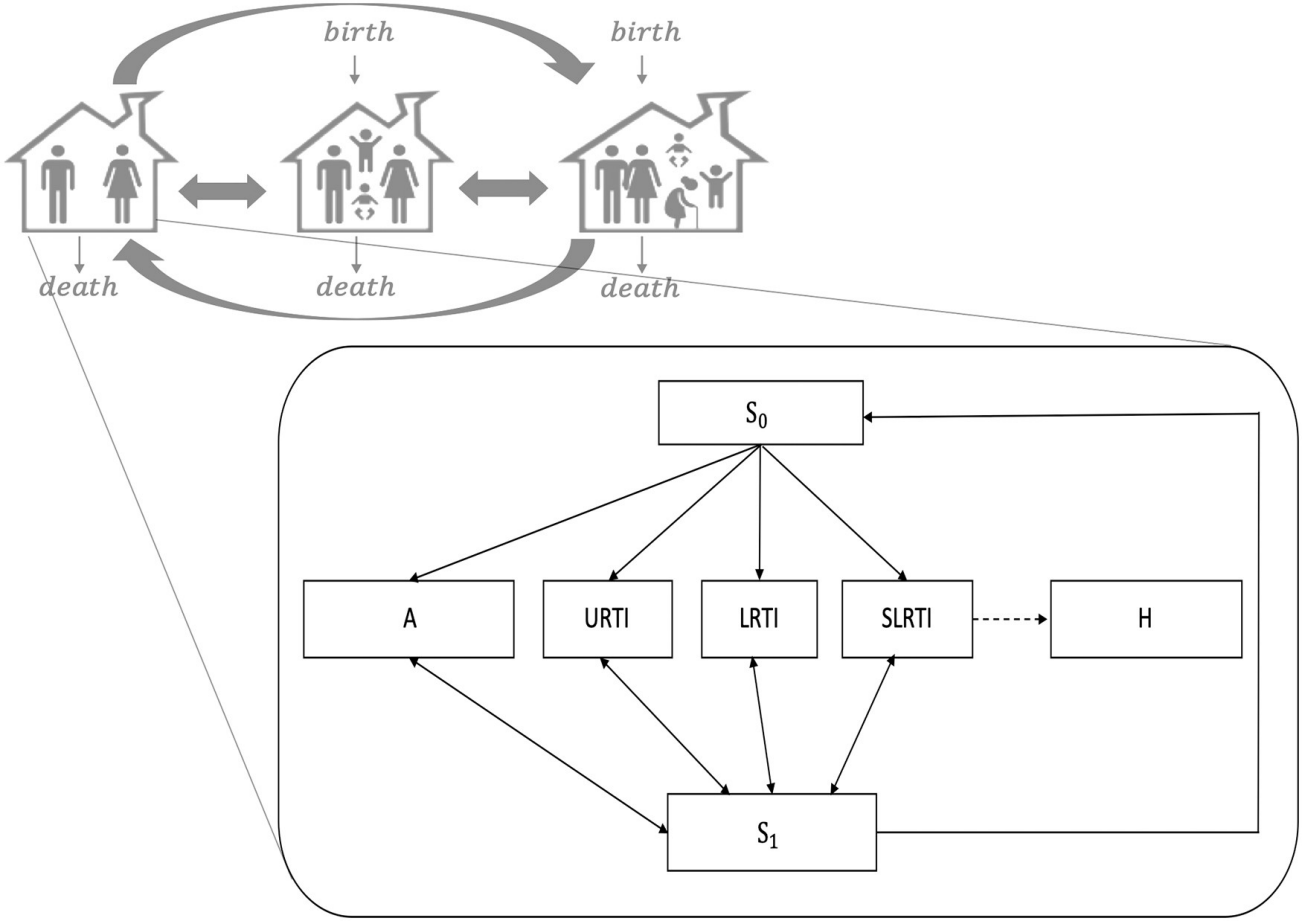
Schematic representation of the RSV transmission dynamic model. Household population were divided into three household types and four age classes: nuclear family type 1 (left), nuclear family type 2 (middle), and extended family type (right). These categories of household population were further divided into six epidemiological compartments as follows: primary susceptible (S_0_), infected but asymptomatic (A), infected and symptomatic categorized as upper respiratory tract infections (URTI), lower respiratory tract infections (LRTI), severe lower respiratory tract infections (SLRTI) (the dashed line represents a proportion of SLRTI who are hospitalized), and secondary susceptible (S_1_) i.e., those still susceptible to infection but with partial immunity.

The household sub-model was then overlaid with the RSV transmission dynamic model, defined in S1 File. The RSV transmission dynamic model (Boosting and waning of immunity model [27]) has its origins in a simple non-age-structured model of RSV dynamics [38]. Then, population in each household type were further divided into six epidemiological compartments, primary susceptible (S_0_), infected but asymptomatic (A), infected and symptomatic categorized as upper respiratory tract infections (URTI), lower respiratory tract infections (LRTI), severe lower respiratory tract infections (SLRTI) and finally, secondary susceptible (S_1_), those still susceptible to infection but with partial immunity (see Fig 1). Hospitalization (H) was considered a subset of SLRTI (see Fig B in S1 File).

RSV-associated ALRI cases by age were obtained from an active population-based surveillance by the Ministry of Public Health, Thailand, in collaboration with the International Emerging Infections Program of the Global Disease Detection, Thailand Regional Center, in two provinces, Sa Kaeo and Nakhon Phanom, since 2003 [19,20]. Geographically, these two provinces represent typical rural provinces on the border; Sa Kaeo is next to Cambodia, while Nakhon Phanom is next to Laos. There was no statistically significant difference in RSV circulation between the two provinces during the study period [19]. Diary contact between age groups are accounted for using a matrix of contact patterns (the “mixing matrix”) empirically derived from a diary-based survey in 2009 [39]. Key assumptions for our model are as follows. First, households were assumed to be of three types (i.e., nuclear family type 1, nuclear family type 2, and extended family type) because these are the ones generally found in Thailand [26]. Second, the household transition rate, defined as the rate at which the household population of each age group changes, was assumed to be consistent over interval times but varying by age group. Third, we assumed that elderly population do not live alone, because elderly people who live alone accounted for only 7% of all elderly population [26]. Fourth, we used fixed diary contact in 2009. Fifth, the immunity status for each person was assumed to be the same and with an absence of any fully resistant person to infection. In this class, people are less likely to be infected and if they are infected, they are less likely to get severe infection. Sixth, maternal immunity was assumed to be absent, according to previous estimations that show the short duration of maternal immunity [27]. Seventh, immunity can wane and secondary susceptible individuals in S_1_ could return to being completely naive susceptible (S_0_). For simplicity, tertiary and subsequent infections were all classified as being secondary infections. Finally, RSV-related deaths were assumed sufficiently few not to affect population and infection dynamics, i.e., negligible for the modeling purpose, and hence they are not explicitly represented in the model equations.

We used R software version 3.3.3 to run and analyze the model outputs and the deSolve package to solve differential equations [40]. The initial parameter values were calculated from a previous study [27]. Model fitting was conducted using the Markov Chain Monte Carlo (MCMC), implemented in the Bayesian Tools R package defined in The Bayesian framework (see S2 File) [41]. For caliberation, the household deterministic sub-model was run from 1995 by fitting the number of population in each household type to the actual population structure of Thailand between 2005 and 2011, according to the Population and Housing Census [34,35] and features of Thai families [26]. We estimated the household transition rates over intervals and used them to run the RSV transmission dynamic model from 1995. The household sub-model together with the RSV transmission dynamic model was run from 1995 to calibrate RSV incidence cases in each household. The RSV transmission dynamic model was then run and fitted to RSV-associated ALRI cases by age group and months from 2005 to 2011 for Sa Kaeo and Nakhon Phanom provinces [19,20]. For model fitting, the DEzs method in the Bayesian Tools package allowed automatic parallelization on three cores to be used for sampling. This method allowed fewer chains to be used for estimating a large number of parameters and thus optimized the computational time [42]. Number of iterations and burn-in were decided upon the model convergence by analyzing the differences between multiple Markov chains. The convergence was assessed by several measures including the standard Gelmal-Rubin procedure [43,44] and the target acceptance rates [45]. Six parameters were estimated including those representing the seasonal parameters (amplitude (*A*), phase angle (*φ*), and Infectivity parameter); within nuclear family type 1 (*q*_*i*1_), within nuclear family type 2 (*q*_*i*2_), and within extended family (*q*_*i*3_) and infectivity in community (*q*_*o*_) (see Table A in S1 File). The median values and credibility intervals were reported. The model was used to assess the impact of household changes in RSV in rural Thailand, sampling all six parameters from the posterior chains and was further used to predict the 10-year RSV incidence in each household type in Thai rural settings.

## Results

The household sub-model was able to calibrate the past population structure of each age group of Thailand for each household type from 2005 to 2011 (see Fig A in S2 File). It was then able to predict the age group population for each household type from 2005 to 2020 (see Fig B in S2 File), and the percentage of population for each household type from 2005 to 2020 (see Fig C in S2 File). The population changes, including household transition rate and death rate modeled here, have a marked effect on the household population structure. The proportion of extended families in the population has been increasing by about 10% and has become a dominant family type, especially elderly population. The classic nuclear family type with parents and children has been declining by about 10%, while nuclear families comprising a husband and wife or single without children have slightly increased over time (see Fig C in S2 File).

**Fig 2:**
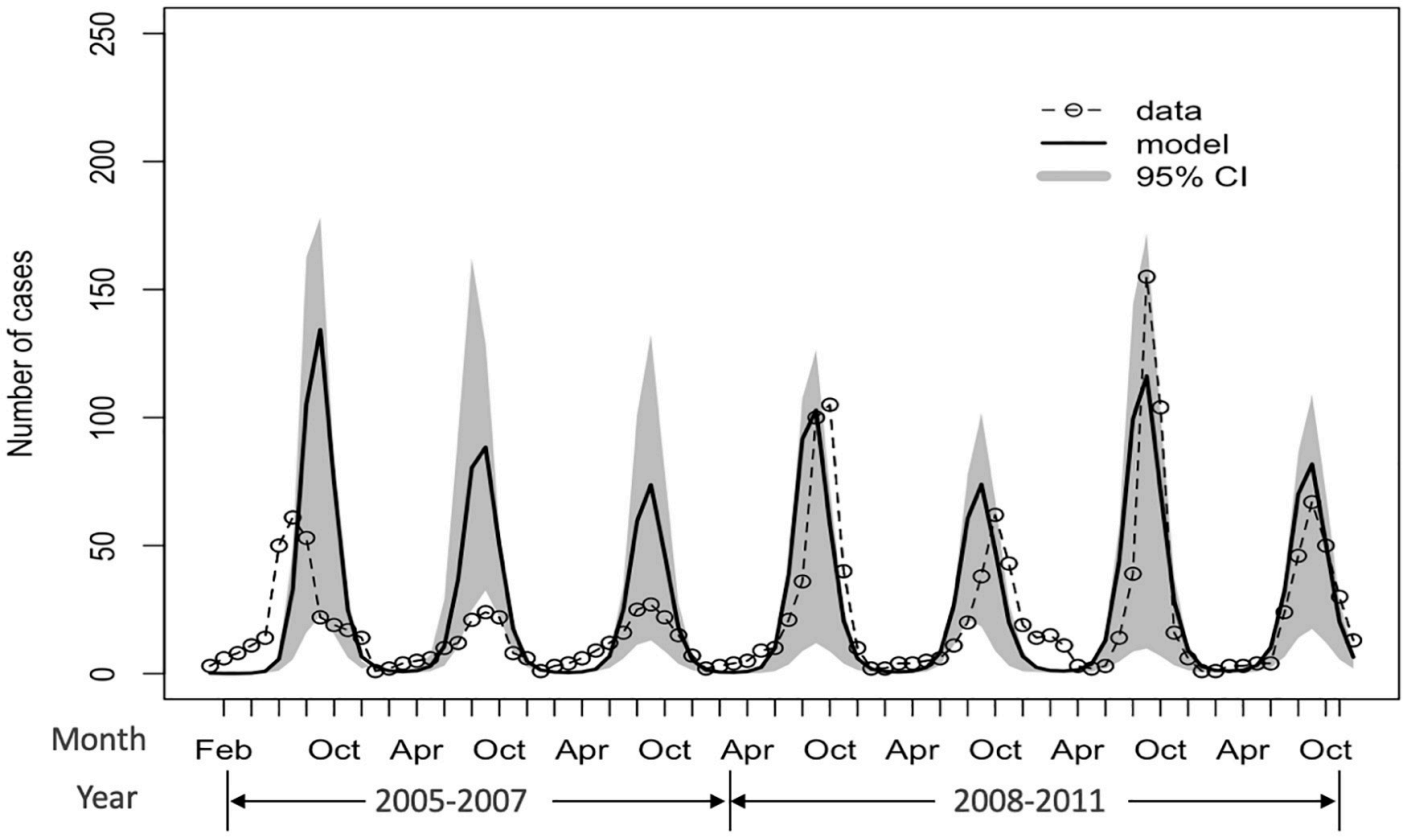
Observed RSV-associated ALRI hospitalizations and the fitted RSV model with 95% CI each month between 2005 and 2011.

The model fitting result is shown in Fig 2. RSV incidence cases occurred seasonally with a peak in the wet season, usually lasting from May to October. The transmission parameters that minimized the fit statistic using the Bayesian method are shown in Table 1. The highest infectivity was estimated among the extended family population, while the lowest was among people in nuclear family type 1.

**Table 1:** Results of estimated parameters of RSV model.

Parameter	Symbol	Value (95% Credible Interval)
Transmission parameters		
**Infectivity within nuclear family type 1**	*q*_*i*1_	0.069 (0.004–0.279)
**Infectivity within nuclear family type 2**	*q*_*i*2_	0.115 (0.110–0.118)
**Infectivity within extended family**	*q*_*i*3_	0.135 (0.128–0.137)
**Infectivity in community**	*q*_*o*_	0.128 (0.011–0.338)
Seasonal parameters		
**Amplitude**	*A*	0.358 (0.344–0.366)
**Phase angle**	*φ*	158.5 (155.7–161.7)

**Fig 3:**
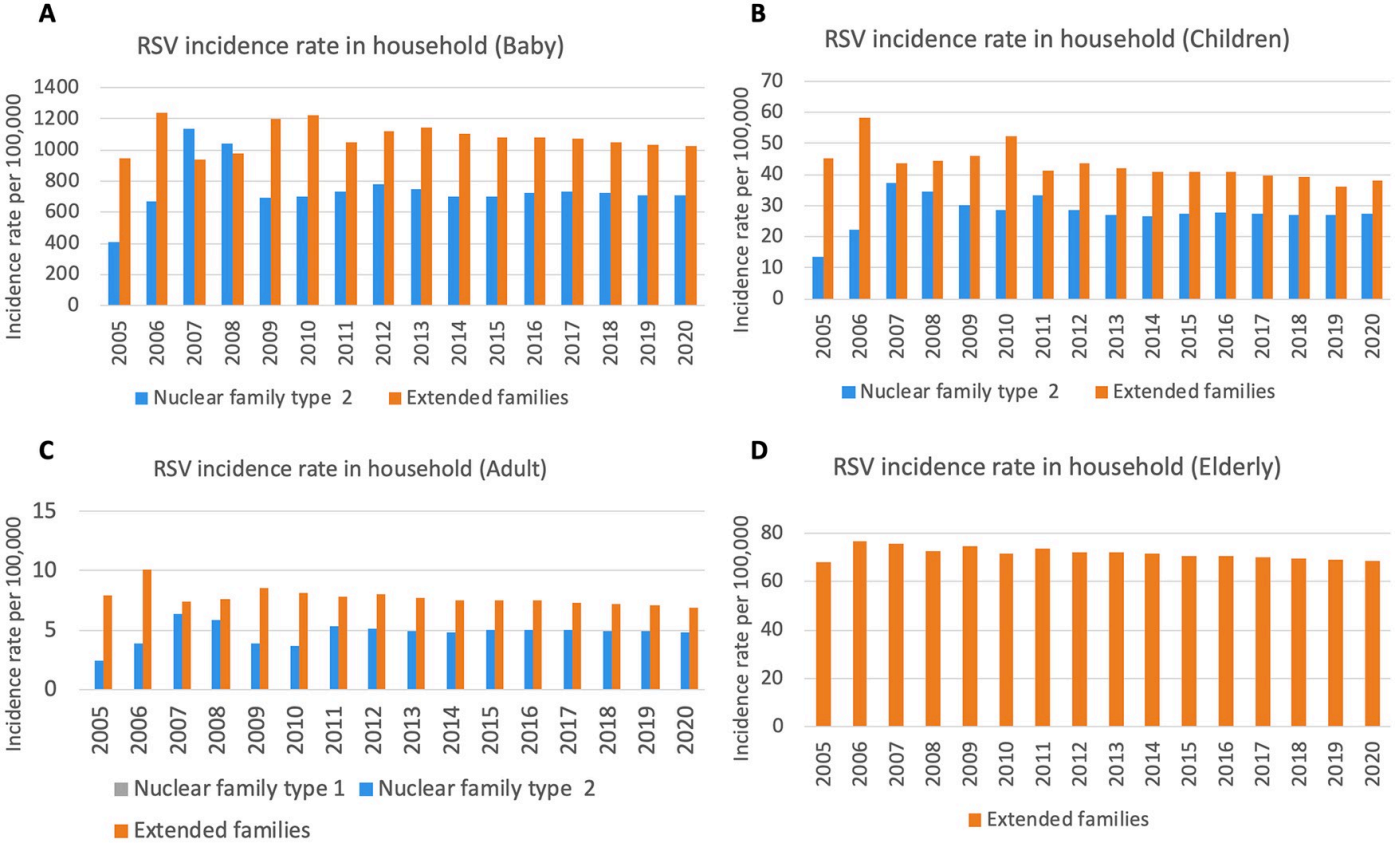
Projection of RSV incidence rate per 100,000 people for each household between 2005 and 2020 in a Thai rural setting: (A) baby, (B) children, (C) adult, and (D) elderly people. Gray color denotes nuclear family type 1, blue color: nuclear family type 2, and orange color: extended family.

Projections of the incidence rates of RSV between 2005 and 2020 are given in Fig 3. The incidence of RSV-associated ALRI hospitalization was predicted to increase from 31.1 (10.7–34.2) cases in 2005 to 35.23 (22.37–38.21) cases in 2011 per 100,000 persons/year. It will decrease by 0.7% in 2020. Incidence rate in the extended family type was two-fold greater than nuclear family type 2. The lowest incidence rate was in the nuclear family type 1. The incidence rate was less than 0.1 per 100,000 and thus unnoticeable in Fig 3C. The majority of RSV incidence rate was estimated to occur among “baby” in the extended family type. It has been decreasing over time after 2013. It will be approximately 864 (845–1,130) cases per 100,000 persons in 2020. The trend of incidence rates among population such as “baby” and “elderly people” was relatively similar.

**Fig 4:**
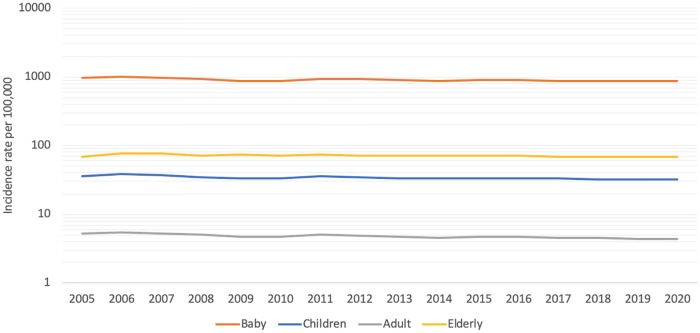
Projection of RSV incidence (logarithmic scale) for each household between 2005 and 2020 in Thai rural setting: Baby (orange color), children (blue color), adult (gray color), and elderly people (yellow color).

We transformed RSV incidence rates into a log scale as showed in Fig 4. The majority of RSV incidence was estimated to occur among the baby population, while RSV incidence among the elderly population was greater than children and adult people. The trend of RSV incidence is steady among populations.

## Discussion

We applied population dynamics and seasonal and household changes to study RSV epidemiology in Thailand. Prior to our study, some statistical analyses were performed to estimate the incidence rate of hospitalizations for RSV-associated ALRI in rural Thai settings between 2004 and 2011 [19,20]. Some similarities and differences between our findings and previously published work are discussed here. Fry and colleagues used a logistic regression model to derive an estimated RSV crude incidence rate of 22 cases per 100,000 population between 2004 and 2007 [19]. Both overall and age-specific incidence rates in our study and their study were lower than our estimations, i.e., overall 30.1 (95% credible intervals, 95% CI 10.7–34.2), baby 1,105.2 (980.3–1,212.1), children 31.5 (20.1–32.8), adult 2.1 (0–4.2), and elderly people 62.6 (42.3–75.2) per 100,000 population. Additionally, our study can predict the RSV incidence among each household type, which has not been accomplished previously in Thailand. Naorat and colleagues used a logistic regression model to derive an estimated total RSV incidence rate of 23 cases per 100,000 from 2008 to 2011 [20]. This figure was lower than our findings, where the overall rate was estimated to be 32.1 (28.2–39.3) cases per 100,000 people, 1,105.4 (980.2–1,212.8)/100,000 for infants, 42.3 (32.1–53.3)/100,000 for children, 3.3 (0–4.2)/100,000 for adults, and 75.3 (50.6–95.1)/100,000 for elderly people. This could partly be explained by the impact of population and household dynamics, as well as a consequence of using different techniques for predictions. Our model tended to fit the observed data well during this period. The model’s prediction of a significantly greater number of cases prior to 2008 could be explained by the screening scheme changes, i.e., before 2008 the eligibility criterion for enrollment was patients with clinician-ordered chest radiographs, so previous estimates did not capture RSV-associated hospitalizations among patients without chest radiographs. Additionally, before 2008, reported RSV diagnoses were made using the RT-PCR technique; after that, rRT-PCR was introduced.

The decreasing trend of RSV incidence rates (0.7%) in these settings was similar to a previous study in the U.S. [46] and some settings in Thailand [47]. Geard and colleagues developed a transmission model and showed that a reduction in fertility rate is associated with a decrease in incidence rate [22]. This finding is similar to our model prediction, although it is important to note that the previous study’s model structure had a Susceptible-Infected-Recovery (SIR) with lifelong immunity following infection; hence, the stronger influence of fertility rate. For a non-immunizing infection such as RSV, where the susceptible pool is also replenished by waning immunity, fertility is likely to have a diminished effect on outbreak dynamics.

Pan-Ngum and colleagues assumed that the infectivity of viral shedding was similar among populations and thus represented by a single estimate [27]. The benefit of using our household model is that it enables us to estimate and compare the infectivity parameter among different household types, which a simpler transmission dynamic model cannot [22,24,27,30]. In our model, we estimated that the infectivity was high among extended family and community. Kombe and colleagues used an individual-level transmission model to derive an estimated figure of the total household incidence rate, which is, in general, higher for larger households due to their potential to have a larger number of infectious individuals at any given time point [33], which is similar to our model estimates. No study has assessed the infectivity at the community level. The high estimate from our model may represent the intense transmission occurring in a school or nursery, for example. Reduction in birth rate over 25 years could reduce the RSV incidence among babies in both nuclear family type 2 (37%) and extended family type (17%), as shown in Fig 3, which is similar to the previous studies [48]. Similarly, the incidence rate among elderly people is also predicted to decrease over time. Yamin and colleagues developed a transmission model study that showed high association of incidence among infants and the elderly, which means that substantial indirect protection targeted at infants and children could reduce RSV in the elderly [49].

Our model can provide insights in terms of RSV in different household types, which can guide health policy planning. Our findings are consistent with some previous studies that estimated RSV incidences were mostly high among babies (92.3%). We also predicted a slight decrease in the RSV incidence among babies while the incidence among children, adults, and the elderly are stably low. However, the estimated incidence rates among elderly people were two-fold greater than those in children. Little attention has been paid to RSV incidence among the elderly in the past, though this will become more significant as the elderly population is rising. Development of concrete strategies to improve standards of care and a potential vaccination program, with a focus on the vulnerable population, will be required. While a vaccine is being developed, some vaccine implementation scenarios could be simulated to identify potential issues around the implementation of such a program and how they intersect with the insights gained using the model. The use of this, or a similar model, to explore some of these control strategies is a clear avenue for future work. Second, the model can be used to guide the design of targeted interventions, i.e., predicting and identifying population at high risk for RSV morbidity. In line with the model’s predictions, targeted intervention strategies could be concentrated on household members of the extended family type. Furthermore, the impact of targeted interventions on infants may yield significant protective benefit to other members of the same household such as elderly people.

Our model has some limitations. First, we used a simple version of the compartmental model structure to study the population and household dynamics and influence on RSV transmission. It might be useful to distinguish between households with children attending day-care or schools compared to children at home. In principle, an individual-based model could capture more fine-grained differences in transmission by household type, e.g., pair-wise contact rates between household members from a survey of primary school students could be considered and potentially be used to parameterize such a model [50]. For simplicity, we assumed that the infectivity, natural mortality rates and transition rates were constant over time, although they varied by age. The transition rates could not be interpreted as evidence for magnitude of these quantities. They served the model fitting purpose such that the household dynamic model could reproduce the actual data. The household structure was assumed fixed beyond 2011 because the survey is performed every 10 years and the next census will be in 2020. In this model, we classified the population of two provinces, namely Sa Kaeo and Nakhon Phanom, representing a Thai rural setting. Scaling-up the model for predicting the RSV disease burden for the whole country might not be appropriate. The population-based surveillance system for clinical pneumonia reports of RSV data used in the model included cases from only eight hospitals in Sa Kaeo Province and twelve hospitals in Nakhon Phanom Province. Additionally, RSV diagnoses were reported differently for two periods, with RT-PCR technique before 2008 and rRT-PCR after 2008, which could, therefore, cause potential inconsistency of prediction.

## Conclusions

Population dynamics, household changes, together with RSV transmission rates, are important components of estimations and predictions of RSV incidence in each household type. The trend of RSV incidence is partly attributable to household changes. The key findings from our study are i) the model predicted the trend of RSV incidence as steady, but it slightly decreased among babies; ii) the infectivity was estimated to be high among the population in extended families; iii) the estimated incidence rates among elderly people were two-fold greater than those in children; and iv) targeted interventions on babies can lessen the transmission to children and the elderly especially when transmission within households of the extended family type is high. Targeted interventions in other age groups may be more complicated, e.g., the case for vaccination of adults or elderly population is cumbersome because boosting pre-existent immunity to RSV may be neutralized by immunosuppressive mechanisms depending on the vaccine characteristics used [51].

We anticipate that the modeling methods described here could be used by other provinces and countries, especially those with reliable census estimates, to estimate future RSV burden, as well as the potential effects of household changes. Additionally, the modeling approach can be adopted to study other infectious diseases and household dynamics considering that demographic factors are important drivers of population health and disease occurrence.

## Supporting information

S1 File Detail of household sub-model and RSV transmission dynamic sub-model. This includes Schematic representation of the household deterministic model (Fig A). Decision tree for distribution of disease states following infection (Fig B). Parameter table for melioidosis transmission dynamic model (Table A). And set of ordinary differential equations for the household sub-model and RSV transmission dynamic sub-model.
(DOCX)

S2 File The Bayesian framework. This includes Bayes theorem, Prior distribution, Likelihood function and Posterior estimation. Number of population by age group in each household type and the fitted household sub-model of the population size each age group and household type of Thai rural settings between 2005 and 2011 (Fig A). Estimation of the age group population each household types of Thai rural settings between 2005 and 2011 (Fig B). Estimation and prediction of the population size each household type of Thai rural settings between 2005 and 2020 (Fig C). Posterior distributions from the melioidosis dynamic model (Fig D).
(DOCX)

## Abbreviations

ALRI: acute lower respiratory tract infection
LRTIs: lower respiratory tract infections
MCMC: Markov Chain Monte Carlo
ODE: Ordinary Differential Equations
rRT-PCR: real-time reverse transcription polymerase chain reaction
RSV: Respiratory Syncytial Virus
RT- PCR: reverse transcriptase polymerase chain reaction assays
