# Supplementary material for: Modeling household dynamics on Respiratory Syncytial Virus (RSV)
Source: PLoS One. 2019 Jul 9;14(7):e0219323. doi: 10.1371/journal.pone.0219323 (PMC6615606; doi:10.1371/journal.pone.0219323)
Supplement: S1 File — This includes Schematic representation of the household deterministic model (Fig A). Decision tree for distribution of disease states following infection (Fig B). Parameter table for melioidosis transmission dynamic model (Table A). And set of ordinary differential equations for the household sub-model and RSV transmission dynamic sub-model. (DOCX) [file pone.0219323.s001.docx]

**S1:** **Detail of household and RSV dynamic models**


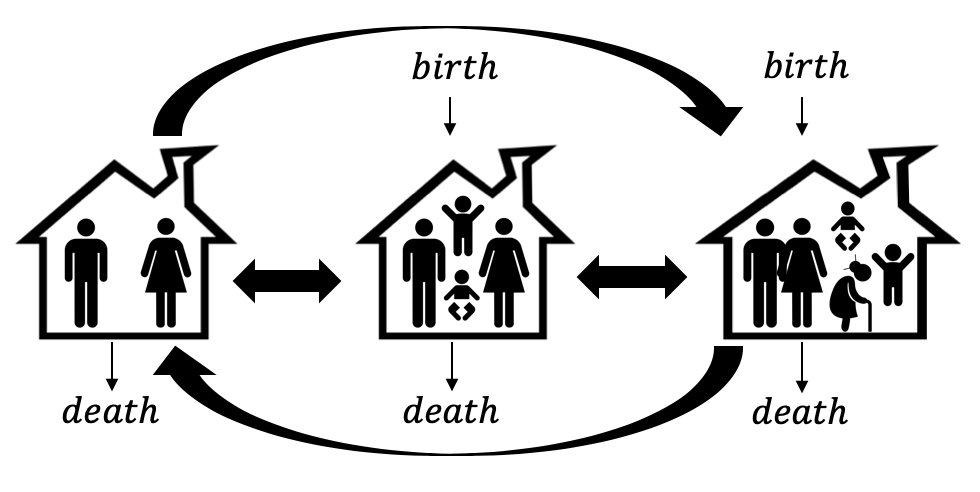


**Fig A.** Schematic representation of the household deterministic model. Household population were further divided into three household types, nuclear family type 1 (left), nuclear family type 2 (middle), and extended family type (right).

**Household sub-model**

We solved a large set of Ordinary Differential Equations (ODE) of Household sub-model. Let $C_{gh}(t)$ be the number of people in each household type *h*, at age group, *g*, at time, *t*. We solved each type of household as follows:

Nuclear family type 1 (Husband and wife or single (aged 15-59 years old) without baby (aged $\leq$ 1) or children (aged 2-14))

Rate of change in Husband and wife or single (adult) without children $C_{a1}$ , was calculated as a balance between the total number of adult $C_{a1}$ and household transition rate of adult (${hr}_{a1})$ and they can die at any time calculated from the adult mortality rate *dr_a_* [1]*:*

$\frac{{dC}_{a1}}{dt}=\left( {hr}_{a1}\times C_{a1} \right)-{dr}_{a}.C_{a1}$ (Equation 1)

Nuclear family type 2 (Husband and wife (aged 15-59) with baby (aged $\leq$ 1) or children (aged 2-14))

Rate of change in the number of adults $(C_{a2}$) in this household was calculated as a balance between the total number of adults $(C_{a2}$) and household transition rate of adult (${hr}_{a2})$ and they can die at any time calculated from the adult mortality rate *dr_a_*. Rate of change in baby population $(C_{b2})$ were calculated from the total number of babies $(C_{b2}$) and household transition rate of baby (${hr}_{b2})$ and baby mortality rate *dr_b_*. Finally, rate of change in the number of children $(C_{c2})$ were then calculated from the total number of children $(C_{c2}$) and household transition rate of children (${hr}_{c2})$ and the child mortality rate *dr_c_*, respectively [1].

$\frac{{dC}_{a2}}{dt}=\left( {hr}_{a2}\times C_{a2} \right)-{dr}_{a}.C_{a2}$

$\frac{{dC}_{b2}}{dt}=\left( {hr}_{b2}\times C_{b2} \right)-{dr}_{b}.C_{b2}$

$\frac{{dC}_{c2}}{dt}=\left( {hr}_{c2}\times C_{c2} \right)-{dr}_{c}.C_{c2}$ (Equation 2)

Extended families type 3 (Three-generation family ((Husband and wife (aged 15-59) with baby (aged $\leq$ 1), children (2-14), and grandparents (aged $\geq$ 60)))

Rate of change in the number of adults $(C_{a3})$) in this household were calculated from the total number of adults $(C_{a3}$) and household transition rate of adult (${hr}_{a3})$ and they can die at any time calculated from the adult mortality rate *dr_a_*. Rate of change in baby population $(C_{b3})$ were calculated from the total number of babies $(C_{b3}$) and household transition rate of baby (${hr}_{b2})$ and baby mortality rate *dr_b_*. Rate of change in the number of children $(C_{c3})$ were calculated from the total number of children $(C_{c3}$) and household transition rate of children (${hr}_{c3})$ and the child mortality rate *dr_c_*. Finally, rate of change in the number of elderly population $(C_{e3})$ were then calculated from the total number of elderly population $(C_{e3}$) and household transition rate of elderly population (${hr}_{e3})$ and the elderly mortality rate *dr_e_* , respectively [1].

$\frac{{dC}_{a3}}{dt}=\left( {hr}_{a3}\times C_{a3} \right)-{dr}_{a}.C_{a3}$

$\frac{{dC}_{b3}}{dt}=\left( {hr}_{b3}\times C_{b3} \right)-{dr}_{b}.C_{b3}$

$\frac{{dC}_{c3}}{dt}=\left( {hr}_{c3}\times C_{c3} \right)-{dr}_{c}.C_{c3}$

$\frac{{dC}_{e3}}{dt}=\left( {hr}_{e3}\times C_{e3} \right)-{dr}_{e}.C_{e3}$ (Equation 3)

All the parameters included in the model was shown in table 1;

Table A Parameter table for RSV transmission dynamic model

| **Parameter** | **Symbol** | **Value**  **(95% Credible Interval)** | **Source/**  **Reference** |
| --- | --- | --- | --- |
| **Population parameters** | | | |
| Mortality rate (${10}^{-6})$ by age group (per capita per day) in 2005 | ${dr}_{b}$ ${dr}_{c}$ ${dr}_{a}$ ${dr}_{e}$ | 49.312  2.38  9.46  140.14 | Census data  [2] |
| Mortality rate (${10}^{-6})$ by age group (per capita per day) in 2009 | ${dr}_{b}$ ${dr}_{c}$ ${dr}_{a}$ ${dr}_{e}$ | 29.52  9.43  11.01  132.12 | Census data  [2] |
| Mortality rate (${10}^{-6})$ by age group (per capita per day) in 2011 | ${dr}_{b}$ ${dr}_{c}$ ${dr}_{a}$ ${dr}_{e}$ | 24.21  7.57  9.45  122.45 | Census data  [2] |
| Household transition rate (${10}^{-6})$ of adult in household type 1 (per capita per day) | ${hr}_{a1}$ In 2005  In 2009  In 2011 | 35.1 (32.3 – 37.8)  51.8 (42.0 – 60.4)  80.3 (66.8 – 93.8) | Estimated |
| Household transition rate (${10}^{-6})$ of adult in household type 2 (per capita per day) | ${hr}_{a2}$ In 2005  In 2009  In 2011 | 30.7 (30.2 – 31.4)  0.03 (0.001 – 0.15)  0.07 (0.001 – 0.39) | Estimated |
| Household transition rate (${10}^{-6})$ of baby in household type 2 (per capita per day) | ${hr}_{b2}$ In 2005  In 2009  In 2011 | 15.7 (10.6 – 19.3)  0.77 (0.01 – 2.97)  2.8 (0.1 – 11.9) | Estimated |
| Household transition rate ${(10}^{-6})$ of children in household type 2 (per capita per day) | ${hr}_{c2}$ In 2005  In 2009  In 2011 | 0.004 (0.001 – 0.02)  0.0072 (0.001 – 0.03)  0.029 (0.001 – 0.19) | Estimated |
| Household transition rate ${(10}^{-6})$ of adult in household type 3 (per capita per day) | ${hr}_{a3}$ In 2005  In 2009  In 2011 | 34.1 (32.83 – 35.5)  65.6 (58.4 – 71.7)  61.2 (50.3 – 79.3) | Estimated |
| Household transition rate ${(10}^{-6})$ of baby in household type 3 (per capita per day) | ${hr}_{b3}$ In 2005  In 2009  In 2011 | 26.2 (25.0 – 36.4)  90.9 (47.2 – 94.9)  55.4 (8.55 – 59.9) | Estimated |
| Household transition rate ${(10}^{-6})$ of children in household type 3 (per capita per day) | ${hr}_{c3}$ In 2005  In 2009  In 2011 | 0.08 (0.01 - 0.29)  45.4 (39.9 – 52.2)  20.9 (8.0 – 39.8) | Estimated |
| Household transition rate ${(10}^{-6})$ of elderly people in household type 3 (per capita per day) | ${hr}_{e3}$ In 2005  In 2009  In 2011 | 234.4 (232.5 – 237.8)  212.7 (201.8 – 221.5)  260.5 (237.6 – 278.2) | Estimated |
| **Contact parameters** | | | |
| Diary contact inside household per person among each age group in 2009 | $c_{b,b}^{i}$,  $c_{b,c}^{i}$,  $c_{b,a}^{i}$, $c_{b,e}^{i}$ | 0.156  0.611  2.572  0.405 | Diary-based survey  [3] |
| Diary contact outside household per person among each age group in 2009 | $c_{b,b}^{o}$,  $c_{b,c}^{o}$,  $c_{b,a}^{o}$, $c_{b,e}^{o}$ | 0.037  0.601  1.208  0.126 | Diary-based survey  [3] |
| **Transmission parameters** | | | |
| Infectivity within nuclear family type 1 | $q_{i1}$ | 0.069 (0.004 - 0.279) | Estimated |
| Infectivity within nuclear family type 2 | $q_{i2}$ | 0.115 (0.110 - 0.118) | Estimated |
| Infectivity within extended family | $q_{i3}$ | 0.135 (0.128 – 0.137) | Estimated |
| Infectivity in community | $q_{o}$ | 0.128 (0.011 – 0.338) | Estimated |
| Rate of recovery from A, URTI per capita per day | $\gamma_{A}$, $\gamma_{U}$ | 0.25 | [4, 5] |
| Rate of recovery from LRTI, SLRTI per capita per day | $\gamma_{L}$, $\gamma_{SL}$ | 0.11 | [4, 5] |
| **Immunity parameters** | | | |
| Immunity factor reducing the susceptibility of previously exposed individuals in $S_{1}$ | $\sigma$ | 0.54 | [6] |
| Rate of waning of short-term immunity of recovered individuals per year | $\rho$ | 0.49 | [7-9] |
| Factor reducing infectiousness of: SLRTI, LRTI, URTI, A | $\alpha_{A}$,  $\alpha_{U}$,  $\alpha_{L}$, $\alpha_{SL}$ | 0.20 (0.17-0.22)  0.45 (0.43-0.48)  0.72 (0.7-0.74)  1 (Fixed) | [10] |
| **Seasonal parameters** | | | |
| Amplitude | $A$ | 0.358 (0.344-0.366) | Estimated |
| Phase angle | $\varphi$ | 158.5 (155.7-161.7) | Estimated |
| Pointiness of the curve | $pt$ | 1 | [10] |

**RSV transmission dynamic sub-model**

Thailand age-specific contact rates

The matrix of contact patterns (the “mixing matrix”) was empirically derived from a diary-based survey in 2009 [3]. In this study, we evaluated the average number of physical contacts inside and outside household that individuals in age group *i* (baby, children, adult, elderly) make with individuals in age group *j* (baby, children, adult, elderly) per day, then corrected for the age group specific proportion of population who participated in the survey i.e. sampling weights, this is then denoted by $c_{ij}$. Then we accounted for the different transmissibility based on the household types i.e. $\beta_{gh,gh}=q_{h}c_{ij}$ , where $q_{h}$ is a fixed fraction which measured the disease specific infectivity of household type *h*. $q_{h}$ were estimated by the model fitting.

Seasonal forcing

The contact rate by age groups (baby, children, adult, elderly), *g*, at household type, *h*, denoted as $\beta_{gh,gh}$, was then used in the models to estimate the force of infection, that is, per susceptible rate of infection. In the absence of a definitive knowledge of the drivers of RSV seasonal patterns, seasonality is modeled using a simple cosinusoidal function. The force of infection by age groups (baby, children, adult, elderly), *g*, at household type, *h* is expressed as:

$$\lambda_{0,gh}\left( t \right)=\sum_{j=gh}^{n} (\frac{\beta_{gh,gh}^{i}\left( A\cos\left( 2\pi\left( t-\varphi\right)+1 \right)^{pt+1-A} \right)}{N_{gh}\left( t \right)})\sum_{k=\left\{ A,U,L,SL,H \right\}} \propto_{k}I_{k,gh}^{i}(t))+\sum_{j=gh}^{n} (\frac{\beta_{gh,gh}^{o}\left( A\cos\left( 2\pi\left( t-\varphi\right)+1 \right)^{pt+1-A} \right)}{N_{gh}\left( t \right)})\sum_{k=\left\{ A,U,L,SL,H \right\}} \propto_{k}I_{k,gh}^{o}(t))$$

$$\lambda_{1,gh}(t)=\sum_{j=gh}^{n} (\frac{\left( 1-\sigma\right)\beta_{gh,gh}^{i}\left( A\cos\left( 2\pi\left( t-\varphi\right)+1 \right)^{pt+1-A} \right)}{N_{gh}\left( t \right)})\sum_{k=\left\{ A,U,L,SL,H \right\}} \propto_{k}I_{k,gh}^{i}(t))+\sum_{j=gh}^{n} (\frac{\left( 1-\sigma\right)\beta_{gh,gh}^{o}\left( A\cos\left( 2\pi\left( t-\varphi\right)+1 \right)^{pt+1-A} \right)}{N_{gh}\left( t \right)})\sum_{k=\left\{ A,U,L,SL,H \right\}} \propto_{k}I_{k,gh}^{o}(t))$$

(Equation 6)

where $\beta_{gh,gh}$ represents the contact matrix (the transmission coefficient between susceptible and infected people inside and outside household), $N_{gh}(t)$ is the total number of infected people inside and outside household at time t, $\propto_{k}I_{k,gh}$, is the relative infectiousness inside and outside household of infected individuals in different infected classes *k (k* = asymptomatic (A), upper respiratory tract infection (URTI), lower respiratory tract infection (LRTI), severe lower respiratory tract infection (SLRTI) and hospitalization (H). Immunity factor reducing the susceptibility of previously exposed individuals in $S_{1}$, $\sigma$. The seasonal parameters defining the relative amplitude, $A$, and the timing of the peak in transmission, $\varphi$, are unknown and are determined by fitting the model to hospitalization data (see detail in *Supporting information file 2*).

Disease stages and severity

The distribution of disease in those infected was assumed to be dependent upon the prior immune status. The distribution follows the decision tree shown in Fig B, such that disease of increasing severity is always a subset of the preceding disease severity group.


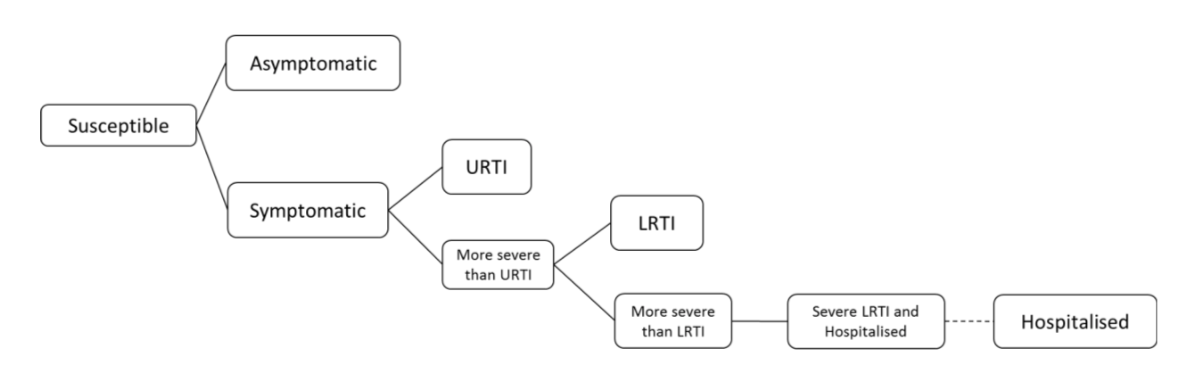


**Fig B.** Decision tree [10] for distribution of disease states following infection. Solid lines represent the nested compartments within each other sequentially and the dashed line represents a proportion of severe LRTI who are hospitalized.

In the model, the host population is stratified into six epidemiological compartments shown in Fig 1 in the main text: primary susceptible (S0), infected but asymptomatic (A), infected and symptomatic categorized as upper respiratory tract infections (URTI), lower respiratory tract infections (LRTI), severe lower respiratory tract infections (SLRTI), or hospitalized (H), and, finally, secondary susceptible (S1), that is, those still susceptible to infection, but who have partial immunity. The proportions of individuals entering each class upon infection are dependent on immune status whose roles was to reduce the susceptibility to subsequent infections as well as the probability of getting the severe forms i.e. LRTI and SLRTI for example. The diagram shows the flow of individuals through the epidemiological compartments. The infection classes i.e. SLRTI, LRTI, URTI, and A are nested within each other sequentially (see Fig B and decision state section above.) This excludes H which is not a separate infection class but a proportion of the SLRTI class. The model includes age group-dependent processes, such as the force of infection and age-group parameters. Thus, all the state variables are stratified by both age group and time such that $S_{0,a}(t)$ represents the density of the primary susceptible of adult (aged 15-59) *a* at time t and so forth. The rates, with respect to both time and age group, at which individuals flow from one epidemiological state to another are described a system of ordinary differential equations.

RSV model equations for each age group *g* in each household type *h* are as follow:

$$\frac{dS_{0,gh}}{dt}=\rho S_{1,gh}-S_{0,gh}\lambda_{0,gh}+{(h}_{gh}\times N_{gh})-{dr}_{g}.S_{0,gh}(t)$$

$$\frac{dI_{A,gh}}{dt}=\lambda_{0,gh}{(1-{SYMPTOM}_{0,gh})S}_{0,gh}+\lambda_{1,gh}(1-{SYMPTOM}_{1,gh})S_{1,gh}-I_{A,gh}\gamma_{A}-{dr}_{g}.I_{A,gh}(t)$$

$$\frac{dI_{U,gh}}{dt}=\lambda_{0,gh}{{SYMPTOM}_{0,gh}(1-LRTI}_{0,gh})S_{0,gh}+\lambda_{1,gh}{SYMPTOM}_{1,gh}(1-{LRTI}_{1,gh})S_{1,gh}-I_{U,gh}\gamma_{U}-{dr}_{g}.I_{U,gh}(t)$$

$$\frac{dI_{L,gh}}{dt}=\lambda_{0,gh}{{SYMPTOM}_{0,gh}LRTI}_{0,gh}(1-{SEVERE}_{0,gh})S_{0,gh}+\lambda_{1,gh}{SYMPTOM}_{1,gh}{LRTI}_{1,gh}(1-{SEVERE}_{0,gh})S_{1,gh}-I_{L,gh}\gamma_{L}-{dr}_{g}.I_{L,gh}(t)$$

$$\frac{dI_{SL,gh}}{dt}=\lambda_{0,gh}{{SYMPTOM}_{0,gh}LRTI}_{0,gh}{SEVERE}_{0,gh}S_{0,gh}+\lambda_{1,gh}{SYMPTOM}_{1,gh}{LRTI}_{1,gh}{SEVERE}_{0,gh}S_{1,gh}-I_{SL,gh}\gamma_{SL}-{dr}_{g}.I_{SL,gh}(t)$$

$$\frac{dS_{1,gh}}{dt}=I_{A,gh}\gamma_{A}+I_{U,gh}\gamma_{U}+I_{L,gh}\gamma_{L}+I_{SL,gh}\gamma_{SL}-S_{1,gh}(\lambda_{1,gh}+\rho)-{dr}_{g}.S_{1,gh}(t)$$

where

$$N_{gh}=S_{0,gh}+I_{A,gh}+I_{U,gh}+I_{L,gh}+I_{SL,gh}+S_{1,gh}$$

Rate of change in the incidence cases was analyzed a balance of lower respiratory tract infections (LRTI), and severe lower respiratory tract infections (SLRTI) as following:

$$\frac{d{New}_{1,gh}}{dt}=\lambda_{0,gh}{{SYMPTOM}_{0,gh}LRTI}_{0,gh}{SEVERE}_{0,gh}S_{0,gh}+\lambda_{1,gh}{SYMPTOM}_{1,gh}{LRTI}_{1,gh}{SEVERE}_{0,gh}S_{1,gh}$$

Rate of change in hospitalization cases was analyzed a balance of hospitalized lower respiratory tract infections (LRTI), and severe lower respiratory tract infections (SLRTI) as following:

$$\frac{d{Hosp}_{1,gh}}{dt}={{HOSP}_{0,gh}\lambda}_{0,gh}{{SYMPTOM}_{0,gh}LRTI}_{0,gh}{SEVERE}_{0,gh}S_{0,gh}+{{HOSP}_{0,gh}\lambda}_{1,gh}{SYMPTOM}_{1,gh}{LRTI}_{1,gh}{SEVERE}_{0,gh}S_{1,gh}$$

The assumptions for this model were explained as following: First, there is an absence of any fully resistant to infection state. Instead, individuals move directly into the secondary susceptible class S1 after their infection. In this class, people are less likely to get infected and if they are infected, they are less likely to get severe infection. Second, immunity can wane and secondary susceptible individuals in S1 could return to completely naive susceptible (S0) i.e. if an individual remains unchallenged for a certain length of time, they can return to S0. For simplicity, tertiary and subsequent infections were all classified as being secondary infections.

**References**

1. Leoprapai B. Thailand's Population Thailand: Mahidol university; 2014.

2. MoPH. Public health statistics A.D.2000. Thailand: Ministry of Public health; 2000.

3. Meeyai A, Praditsitthikorn N, Kotirum S, Kulpeng W, Putthasri W, Cooper BS, et al. Seasonal influenza vaccination for children in Thailand: a cost-effectiveness analysis. PLoS Med. 2015;12(5):e1001829; discussion e. Epub 2015/05/27. doi: 10.1371/journal.pmed.1001829. PubMed PMID: 26011712; PubMed Central PMCID: PMCPMC4444096.

4. Hall C, Geiman J, Biggar R, Kotok D, Hogan P, Douglas RJ. Respiratory syncytial virus infections within families. New England Journal of medicine. 1976;294:414-9.

5. Okiro EA, White LJ, Ngama M, Cane PA, Medley GF, Nokes DJ. Duration of shedding of respiratory syncytial virus in a community study of Kenyan children. BMC Infect Dis. 2012;10(1):15. PubMed PMID: 20096106.

6. Henderson FW, Collier AM, Clyde WA, Jr., Denny FW. Respiratory-syncytial-virus infections, reinfections and immunity. A prospective, longitudinal study in young children. N Engl J Med. 1979;300(10):530-4. doi: 10.1056/NEJM197903083001004. PubMed PMID: 763253.

7. Agoti CN, Mwihuri AG, Sande CJ, Onyango CO, Medley GF, Cane PA, et al. Genetic relatedness of infecting and reinfecting respiratory syncytial virus strains identified in a birth cohort from rural Kenya. J Infect Dis. 2012;206(10):1532-41. doi: 10.1093/infdis/jis570. PubMed PMID: 22966119; PubMed Central PMCID: PMCPMC3475639.

8. Ohuma EO, Okiro EA, Ochola R, Sande CJ, Cane PA, Medley GF, et al. The natural history of respiratory syncytial virus in a birth cohort: the influence of age and previous infection on reinfection and disease. Am J Epidemiol. 2012;176(9):794-802. doi: 10.1093/aje/kws257. PubMed PMID: 23059788; PubMed Central PMCID: PMCPMC3481264.

9. Scott PD, Ochola R, Ngama M, Okiro EA, James Nokes D, Medley GF, et al. Molecular analysis of respiratory syncytial virus reinfections in infants from coastal Kenya. J Infect Dis. 2006;193(1):59-67. doi: 10.1086/498246. PubMed PMID: 16323133; PubMed Central PMCID: PMCPMC2384051.

10. Pan-Ngum W, Kinyanjui T, Kiti M, Taylor S, Toussaint JF, Saralamba S, et al. Predicting the relative impacts of maternal and neonatal respiratory syncytial virus (RSV) vaccine target product profiles: A consensus modelling approach. Vaccine. 2017;35(2):403-9. doi: 10.1016/j.vaccine.2016.10.073. PubMed PMID: 27914740; PubMed Central PMCID: PMCPMC5221409.
