# Supplementary material for: Modeling household dynamics on Respiratory Syncytial Virus (RSV)
Source: PLoS One. 2019 Jul 9;14(7):e0219323. doi: 10.1371/journal.pone.0219323 (PMC6615606; doi:10.1371/journal.pone.0219323)
Supplement: S2 File — This includes Bayes theorem, Prior distribution, Likelihood function and Posterior estimation. Number of population by age group in each household type and the fitted household sub-model of the population size each age group and household type of Thai rural settings between 2005 and 2011 (Fig A). Estimation of the age group population each household types of Thai rural settings between 2005 and 2011 (Fig B). Estimation and prediction of the population size each household type of Thai rural settings between 2005 and 2020 (Fig C). Posterior distributions from the melioidosis dynamic model (Fig D). (DOCX) [file pone.0219323.s002.docx]

**S2: The Bayesian framework**

Bayesian inference of household transition and RSV transmission dynamic model provide a framework for estimating parametric uncertainty in terms of probabilistic distributions and allowing a direct quantification of parameter uncertainty.

Bayes theorem states that the best estimate (posterior uncertainty $p(\theta|y$)) for a parameter vector $\theta$ given data y is given by:

$p\left( \theta| y \right)=\frac{p\left( \theta\right)p(y|\theta)}{p(y)}$ (Equation 1)

Here, $p\left( \theta\right)$ is the prior information and*,*$\frac{p(y|\theta)}{p(y)}$ is the likelihood ratio. Markov Chain Monte Carlo *(MCMC)* algorithms were applied to approximate these distributions which used a sampling scheme to estimate the posterior distribution [1].

## Prior distribution

Uniform distribution was chosen to be the prior distribution for all parameter values, given that little information about these parameters has been measured or reported. The minimum and maximum values were initially determined then narrowed down from the iterative model fitting procedure.

## Likelihood function

We defined the likelihood as the product of likelihood terms for each data point. The data arise from the actual population structure of Thailand between 1995 and 2011 from the Population and Housing Census [2, 3], features of Thai families [4] and the RSV epidemiological surveillance report between 2005 and 2011 and are linked to the summation of expected age and gender rates via a Poisson distribution. The log-likelihood function of household deterministic sub-model assuming Poisson distributed data is

${LL}_{house}=\sum_{a} \left( \sum_{t} log\left( \frac{H^{\theta h}exp(-H)}{\theta h!} \right) \right)$ (Equation 2)

Where *θh(a,t)* is the number of population each household age group *g*  time *t* and *H* is the expected number of population by age group each household from the model at each age group *g* and time *t*.

The log-likelihood function of RSV transmission model assuming Poisson distributed data is

${LL}_{RSV}=\sum_{a} \left( \sum_{t} log\left( \frac{{RSV}^{\theta r}exp(-RSV)}{\theta r!} \right) \right)$ (Equation 3)

Where *θr(a,t)* is the incidence of RSV each household at each age group *g* and time *t*, and *RSV* is the expected incidence of RSV each household by model at each age group *g* and time *t*.

## Posterior estimation

We used a Differential Evolution Markov Chain Monte Carlo (MCMCzs, or DE-MCzs) method to estimate the posterior distributions. We considered Markov Chain methods of sampling that were proposed by Ter Braak and Vrugt et al, 2008 [5], which have previously been used for numerical problems, and implemented using the Bayesian Tools R package. Differential Evolution Markov Chain (DE-MC) is an adaptive MCMC algorithm, in which multiple chains are run in parallel and presented. The DE-MCzs method combines characteristics of conventional MCMC methods with the ideas of differential evolution optimization algorithms, by making use of the full joint density function and (independent) proposal distributions for each of the variables including population, household changes, and incidence rate of RSV. These samples are accepted probabilistically based on the acceptance probability. Uniform distributions are centered at the current state of the chain. This proposal distribution randomly perturbs the current state of the chain, and then either accepts or rejects the perturbed value. Two chains ran in parallel on three cores, each consisting of 15,000 iterations and a burn-in period of 3,500 iterations for household model, were run in parallel to achieve a target acceptance rate of 0.2. While, RSV model were run for 2,600 iterations, and a burn-in period of 500 iterations in parallel to achieve a target acceptance rate of 0.2 and are shown in Fig D.


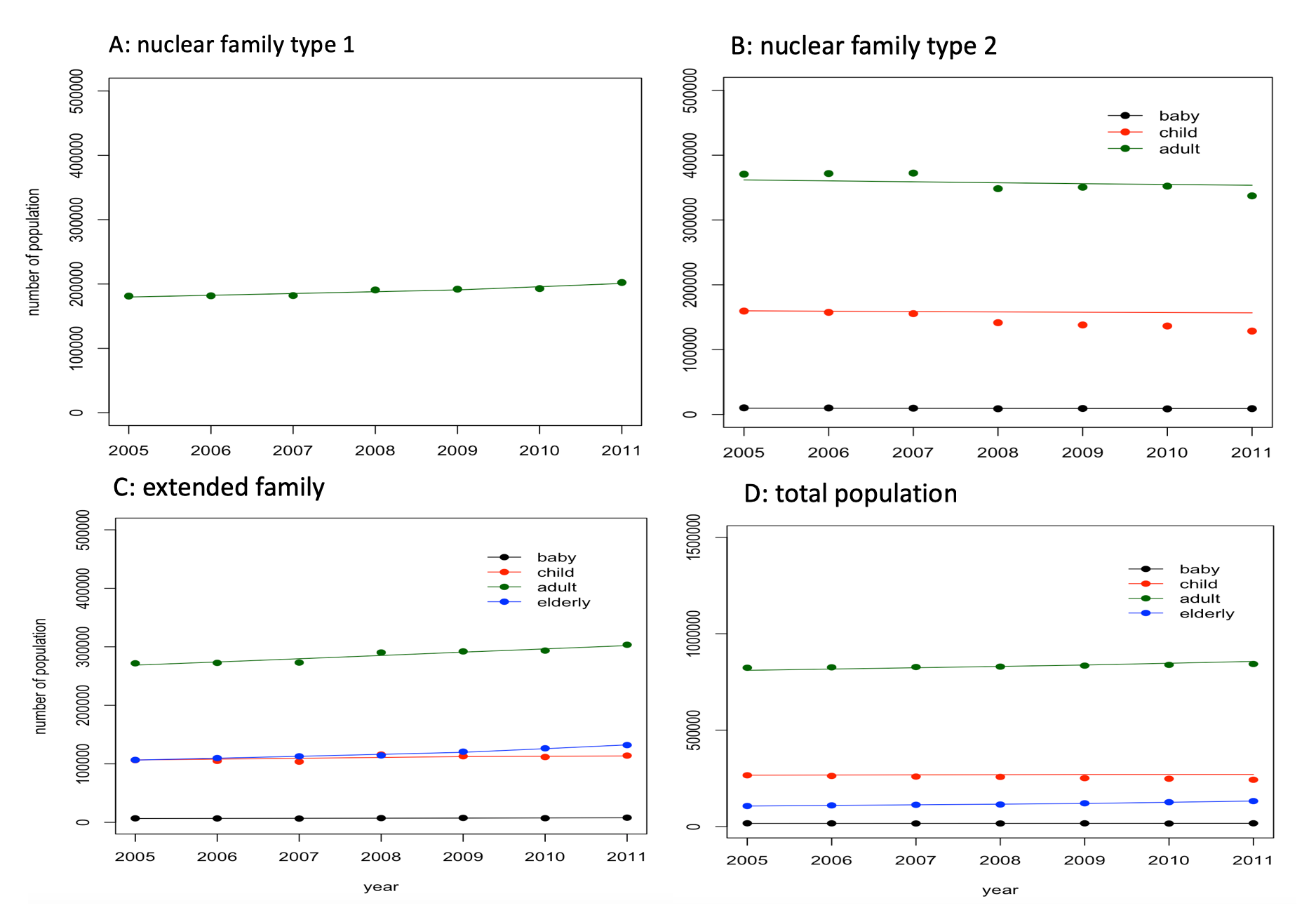


Fig A. Number of population by age group in each household type and the fitted household sub-model of the population size each age group and household type of Thai rural settings between 2005 and 2011. A: nuclear family type 1; B: nuclear family type 2; C: extended family; D: total population. Color dot: total population each year; line: model.


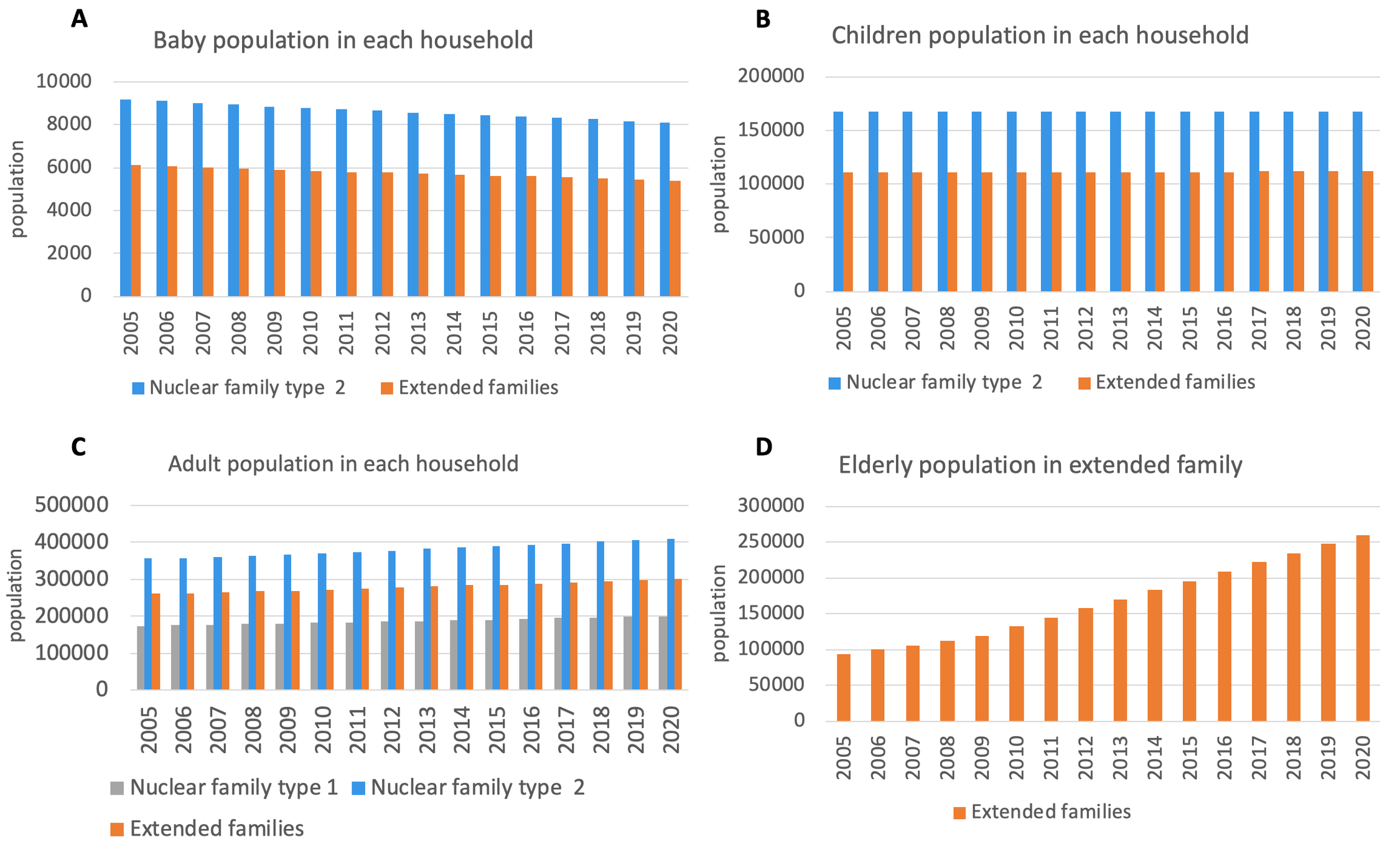


Fig B. Estimation of the age group population each household types of Thai rural settings between 2005 and 2020: (A) Adult (B) Baby (C) Children, and (D) Elderly. Gray: nuclear family type 1; Blue: nuclear family type 2; Orange: extended family type 3.


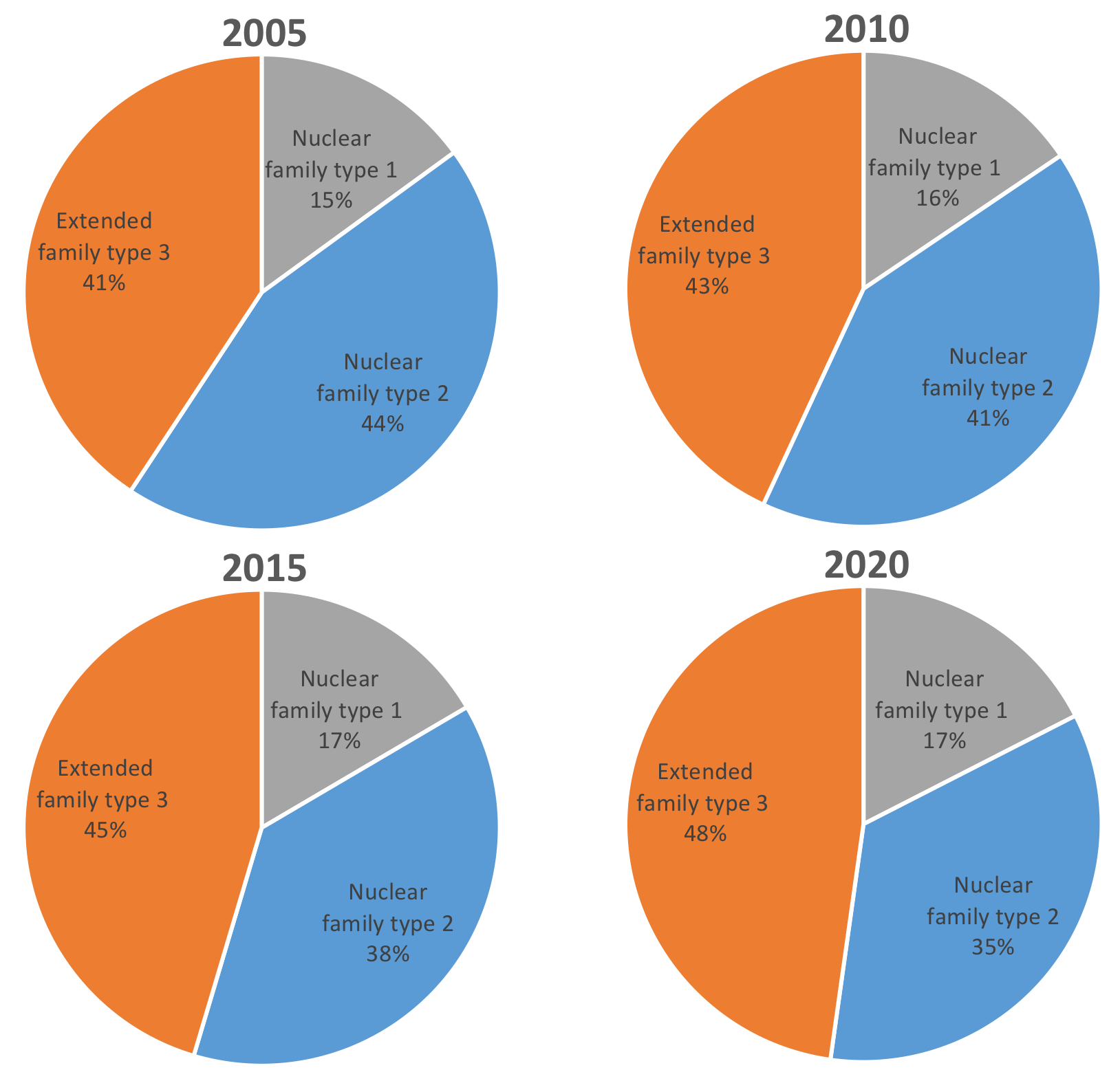


Fig C. Estimation and prediction of the population size each household type of Thai rural settings between 2005 and 2020. Gray: nuclear family type 1; Blue: nuclear family type 2; Orange: extended family type 3.


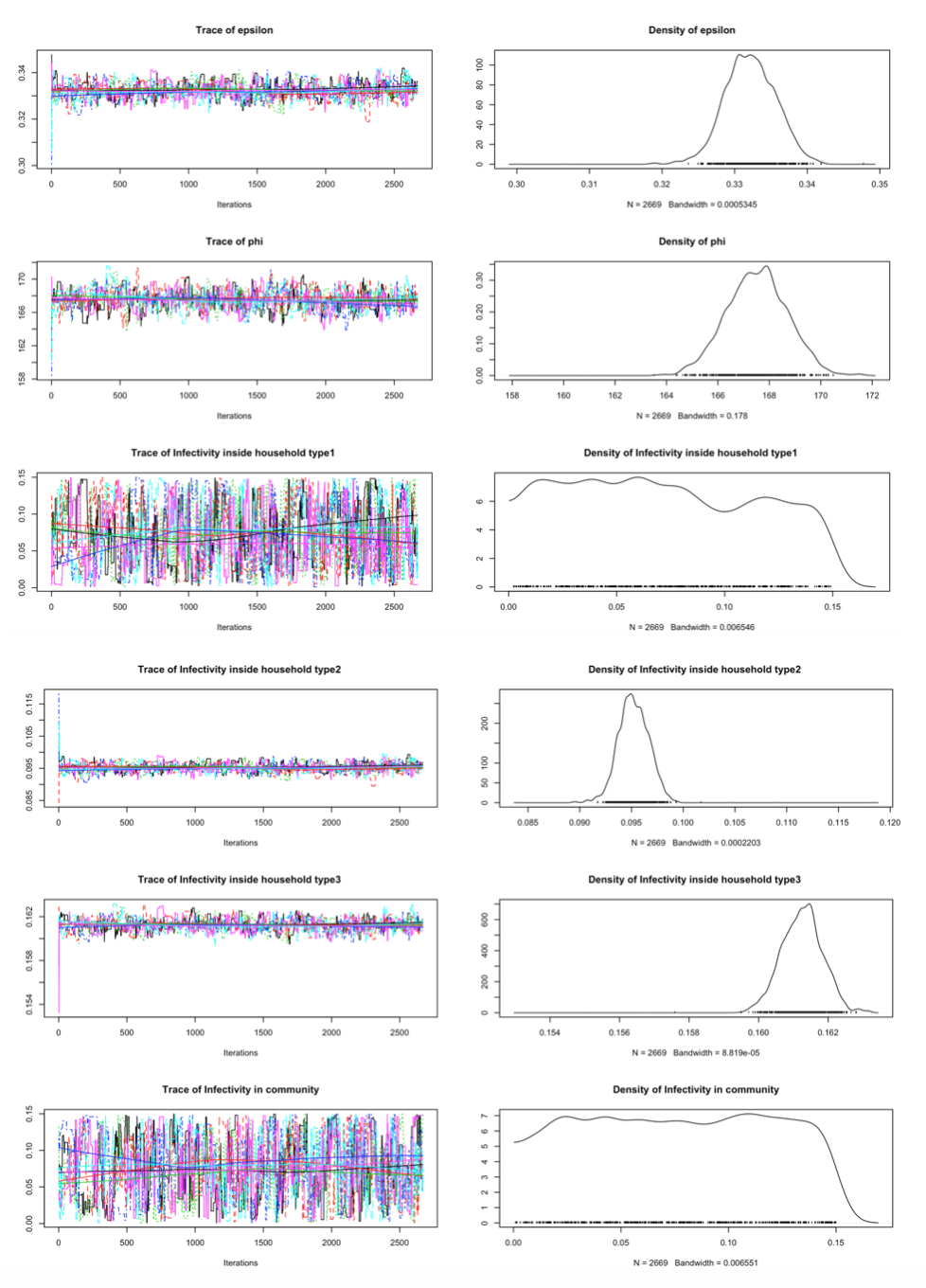


Fig D. Posterior distributions from the RSV dynamic model, that each row corresponds to the separate parameter, the left-hand column contains traces with 6 color chains (dashed lines: each chain, solid lines: trend of each chain) and the right-hand column contains the posterior distribution, corresponding to each parameter.

# References

1. Augustynczik ALD, Hartig F, Minunno F, Kahle H-P, Diaconu D, Hanewinkel M, et al. Productivity of Fagus sylvatica under climate change – A Bayesian analysis of risk and uncertainty using the model 3-PG. Forest Ecology and Management. 2017;401(Supplement C):192-206. doi: <https://doi.org/10.1016/j.foreco.2017.06.061>.

2. USCB. International Data Base United States Census Bureau: United State; 2016 [cited 2016 21 August]. Available from: <https://www.census.gov/population/international/data/idb/informationGateway.php>.

3. NSO. The 2010 population and housing census. Thailand: National statistical office, office of the prime minister; 2010.

4. UNFPA. The State of Thailand’s Population 2015, Features of Thai Families in the Era of Low Fertility and Longevity. Thailand: the United Nations Population Fund Thailand and the Of ce of the National Economic and Social Development Board, 2015.

5. Ter Braak CJ, Vrugt, J.A. Differential evolution Markov chain with snooker updater and fewer chains. Stat Comput 2008;18:435–46.
